# Effects of Combined Simultaneous and Sequential Endostar and Cisplatin Treatment in a Mice Model of Gastric Cancer Peritoneal Metastases

**DOI:** 10.1155/2017/2920384

**Published:** 2017-01-19

**Authors:** Lin Jia, Shuguang Ren, Tao Li, Jianing Wu, Xinliang Zhou, Yan Zhang, Jianhua Wu, Wei Liu

**Affiliations:** ^1^Department of Medical Oncology, The Affiliated Hospital of Hebei University, Baoding 071000, China; ^2^Department of Animal Center, The Fourth Hospital, Hebei Medical University, Shijiazhuang 050017, China; ^3^Department of Epidemiology and Health Statistics, School of Public Health, Hebei Medical University, Shijiazhuang 050011, China; ^4^Hebei Province China-Japan Friendship Center for Cancer Detection, Shijiazhuang 050017, China; ^5^Department of Medical Oncology, The Fourth Hospital, Hebei Medical University, Shijiazhuang 050017, China; ^6^Department of Palliative Care Center, Beijing Cancer Hospital, Beijing 100000, China

## Abstract

*Objective*. Aimed to study the effects of endostar and cisplatin using an in vivo imaging system (IVIS) in a model of peritoneal metastasis of gastric cancer.* Methods*. NUGC-4 gastric cancer cells transfected with luciferase gene (NUGC-4-Luc) were injected i.p. into nude mice. One week later, mice were randomly injected i.p.: group 1, cisplatin (d1–3) + endostar (d4–7); group 2, endostar (d1–4) + cisplatin (d5–7); group 3, endostar + cisplatin d1, 4, and 7; group 4, saline for two weeks. One week after the final administration, mice were sacrificed. Bioluminescent data, microvessel density (MVD), and lymphatic vessel density (LVD) were analyzed.* Results*. Among the four groups, there were no significant differences in the weights and in the number of cancer cell photons on days 1 and 8 (*P* > 0.05). On day 15, the numbers in groups 3 and 1 were less than that in group 2 (*P* < 0.05). On day 21, group 3 was significantly less than group 2 (*P* < 0.05). MVD of group 4 was less than that of groups 1 and 2 (*P* < 0.01). There was no significant difference between groups 2 and 3 (*P* > 0.05) or in LVD number among the four groups (*P* > 0.05).* Conclusions*. IVIS® was more useful than weight, volume of ascites, and number of peritoneal nodules. The simultaneous group was superior to sequential groups in killing cancer cells and inhibiting vascular endothelium. Cisplatin-endostar was superior to endostar-cisplatin in killing cancer cells, while the latter in inhibiting peritoneal vascular endothelium.

## 1. Introduction

Malignant ascites is common in gastrointestinal and gynecological cancers and has been associated with a median survival of less than 20 weeks [1]. Patients with malignant ascites caused by gastrointestinal cancers have especially poor prognosis, and their survival times are only 12–20 weeks [2]. The treatment of malignant effusions is often a challenge for physicians. Currently, the conventional treatment of malignant effusions is mainly composed of diuresis, salt restriction, serous cavity paracentesis, intracavitary chemotherapy, biological response modifiers, traditional Chinese medicine, or thermotherapy. However, these therapies are not all satisfactory. After treatment with these methods, there is no significant decrease in effusions, and relapses often occur. Furthermore, almost all of these treatment methods have toxic side effects to various degrees [3].

Thus, it is important to understand the underlying molecular mechanisms associated with malignant effusion. Previous studies have shown that elevated levels of vascular endothelial growth factor (VEGF), tumor angiogenesis, and increased vascular permeability after tumor invasion or metastasis to the pleuroperitoneum are important mechanisms of serous cavity effusions [4, 5]. VEGF has attracted attention due to its presence in the pleural fluid and its potential use as a therapeutic target [6–8]. Many clinical studies have also demonstrated the potential benefit of inhibition of VEGF-A in patients with malignant effusions [4]. Antiangiogenic therapy (such as bevacizumab, a monoclonal antibody targeting VEGF-A) adjuvant to chemotherapy was found to have a potential role in management of pleural effusion in advanced nonsquamous non-small-cell lung cancer [8].

Recombinant human endostatin (rh-endostatin, endostar) is a Chinese broad spectrum humanized antiangiogenic drug that targets vascular endothelial cells, but not tumor cells. Furthermore, this drug has been shown to downregulate the protecting effect of a variety of proangiogenic factors on the vascular endothelium. In addition, it has direct and indirect antiangiogenic effects. Endostar was approved by the State Food and Drug Administration of China (SFDA) for the treatment of non-small-cell lung cancer in 2005 [9]. A number of researchers have explored the application of endostar alone or in combination with chemotherapy for treatment of malignant serous effusion showing high efficiency and low toxicity. It has been shown that the control of effusions is stronger than other agents and that this can significantly improve quality of life of patients. Using a malignant pleural effusion (MPE) model, it has been demonstrated that endostar had an efficient anticancer activity in MPE through its suppressive effect on angiogenesis and lymphangiogenesis. However, bevacizumab does not inhibit lymphangiogenesis. This provided a theoretical basis for the use of endostar for MPE treatment [10]. However, the efficacy of endostar when administered simultaneously and sequentially with chemotherapy remains to be determined.

In mice subcutaneous tumor models, tumor growth can be easily monitored by caliper measurements. However, in pleural metastasis models, it is difficult to continuously measure tumor growth and evaluate the response to a treatment. Surrogate markers such as weight loss may be employed to monitor toxicity. However, the real treatment efficacy can usually be evaluated only after mice are sacrificed. In addition to computed tomography (CT), magnetic resonance imaging (MRI), and positron emission tomography (PET), an in vivo noninvasive bioluminescent imaging (BLI) system has recently been developed using the adenosine triphosphate- (ATP-) dependent light-emitting reaction of the firefly (*Photinus pyralis*) luciferase and its substrate, D-luciferin [D-(−)-2-(6-hydroxy-2-benzothiazolyl) thiazone-4-carboxylic acid] [11, 12]. Using this system, the temporal and spatial monitoring of the pathophysiological processes can be performed in vivo, thus, reducing the number of animals needed to achieve statistical power [11–18].

We established a stably expressing luciferase gastric NUGC-4-Luc cell line. On the basis of this cell line, we established a gastric cancer ascites tumor model in nude mice for the first time and demonstrated the therapeutic effects of the simultaneous and sequential administration of endostar with cisplatin on the intraperitoneal disseminated foci using an in vivo imaging system (IVIS) and detected the peritoneal nodules with microvessel density (MVD) and lymphatic vessel density (LVD).

## 2. Materials and Methods

### 2.1. Cell Line and Animals

Human gastric adenocarcinoma cell line NUGC-4 was obtained from Japan RIKEN BioResource (Tokyo, Japan). A luciferase-expressing human gastric cancer cell line, NUGC-4-Luc, was established by GenScript Co., Ltd. (Nanjing, China). Cells were maintained at 37°C in RPMI-1640 medium (Gibco, Grand Island, NY) supplemented with 10% fetal bovine serum at 37°C in a humidified atmosphere containing 5% CO_2_. Five- to six-week old female nude mice (BALB/c nu/nu) were purchased from the Institute of Laboratory Animal Science, the Chinese Academy of Medical Sciences, Beijing, China, and were housed in environmentally controlled conditions (22°C, 12-hour light/dark cycles, with the light cycle from 6:00 to 18:00 and the dark cycle from 18:00 to 6:00) with ad libitum access to standard laboratory chow. The study protocol was approved by the local Institutional Review Board, and animal experiments were conducted in accordance with the guideline of the local Institutional Animal Care and Use Committee, which has been accredited by the Association for Assessment and Accreditation of Laboratory Animal Care International.

### 2.2. Ascites Tumor Model and Therapeutic Experiments

In the present study, stable integrated luciferase gastric undifferentiated NUGC-4-Luc cell lines were established. Then, cells were intraperitoneally injected into nude mice to produce the gastric cancer ascites tumor model in 3–5 days. NUGC-4-luc cells were cultured in RPMI1640 medium + 10% FBS + 1% P/S and cell suspension for ultimate collection was a total of 5 × 10^6^ NUGC-4-luc cells in 50 mL of PBS. The cell suspension was injected into the abdominal cavities of 28 female nude mice [19–21]. These mice underwent in vivo bioluminescence imaging weekly. One week after NUGC-4-Luc cell injection, mice were randomly divided into four groups with seven mice each and were injected intraperitoneally with the following: group 1, cisplatin (purchased from QiLu Pharmaceutical Co., Shandong, China; 1 mg/kg) at days 1–3 + endostar (purchased from Simcere Pharmaceutical Co., Jiangsu, China; 8 mg/kg) at days 4–7; group 2, endostar (8 mg/kg) at days 1–4 + cisplatin (1 mg/kg) at days 5–7; group 3, endostar (8 mg/kg) + cisplatin (1 mg/kg) at days 1, 4 and 7; group 4 (control group), 50 *μ*L of normal saline at days 1–7 [10, 22]. Each group was treated for two consecutive weeks and underwent imaging weekly. One week after the final administration, mice were anesthetized and sacrificed. Peritoneal metastasis of gastric carcinoma was monitored with IVIS.

### 2.3. Bioluminescence Imaging with IVIS

Mice were anesthetized by isoflurane inhalation and were subsequently injected i.p. with 100 *μ*L of 7.5 mg/mL of D-luciferin (Xenogen). Bioluminescence imaging with a CCD camera (IVIS, Xenogen) was initiated 10 min after injection. Imaging times ranged from 1 to 60 sec, depending on the amount of luciferase activity. Bioluminescence from the region of interest (ROI) was defined manually, and the data were expressed as photon-flux (photons/s/cm^2^/steradian). All bioluminescent data were collected and analyzed using IVIS [23].

### 2.4. Immunohistochemical Staining and Evaluation

Tumor tissue samples were fixed with 10% buffered formalin and embedded in paraffin after routine dehydration. Consecutive 5 *μ*m sections were cut from each block, immunostained, and analyzed for MVD and LVD. Tissue sections were stained with anti-CD34 antibody (rabbit anti-mouse rat anti-mouse monoclonal antibody, 1 : 200; eBioscience, US) and anti-D2-40 antibody (rat anti-mouse monoclonal antibody, 1 : 200; Upstate, US). Biotinylated anti-rat or rabbit antibodies (Beijing Zhongshan Golden Bridge Biological Technology CO., Beijing, China) were used as secondary antibodies. Staining for CD31 was used to evaluate MVD, which were assessed by counting all stained vessels at 6,200x magnification. The mean number of vessels was defined as MVD [19]. Staining for D2-40 was used to evaluate LVD, and the same method was used to evaluate MVD. The stained slides were reviewed and scored independently by two investigators (Drs. Wu and Zhang), who were blinded to the slide identification and clinical data. Disagreements were resolved by discussion to reach a consensus.

### 2.5. Statistical Analysis

Statistical analysis was performed using the SPSS software package (version 17.0; SPSS Inc., Chicago, IL, USA). Bioluminescence imaging data from IVIS and weights of the nude mice were analyzed by repeated measurements. Analysis of variance in repeated measurement data and comparison of data between various groups were performed by multivariate analysis. One-way analysis of variance (ANOVA) was used to assess the statistical significant differences in MVD among groups. Comparisons of LVD among various groups were performed using a nonparametric Kruskal-Wallis test, followed by the Mann–Whitney test. All *P* values were two-tailed. *P* values < 0.05 were considered significant.

## 3. Results

### 3.1. Ascites Tumor Model

**Figure 1:**
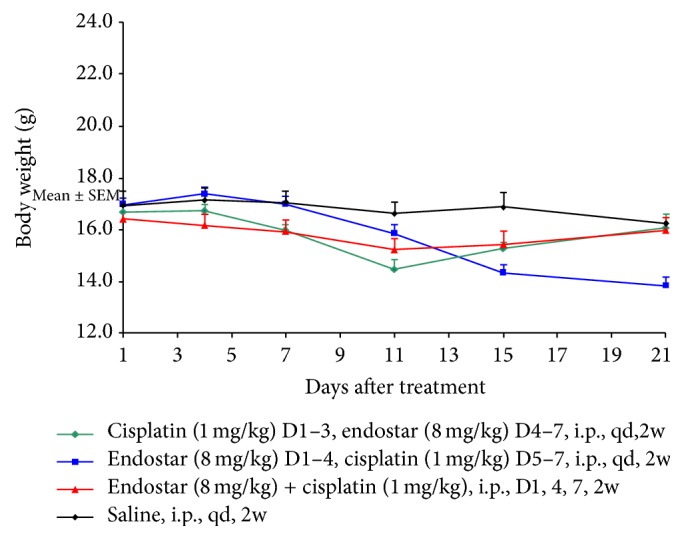
The differences in weight changes in nude mice among the four treatment groups as presented by the repeated measures process of the general linear model.

The background number of cancer cell photons prior to treatment was not significantly different among the four groups (*P* > 0.05). During the treatment, one mouse in the control group died. All of the other mice survived until the experiment was completed. There were no significant differences in nude mice weights between the four treatment groups (*P* > 0.05, Figure 1). After sacrifice, bloody ascites and the small, numerous, and widely distributed peritoneal nodules were observed.

### 3.2. Drug Efficacy

The group rank of the volume (mL) of ascites in four groups was as follows: group 4 (4.87 ± 0.45) > group 1 (3.1 ± 0.53) > group 2 (2.0 ± 0.08) > group 3 (1.8 ± 0.16) (*P* < 0.05). The group rank for the number of peritoneal nodules was as follows: group 4 (33.75 ± 2.5) > group 2 (21.66 ± 5.77) > group 3 (18.75 ± 2.5) > group 1 (8.75 ± 4.78) (*P* < 0.05).

### 3.3. The Number of Photons

**Figure 2:**
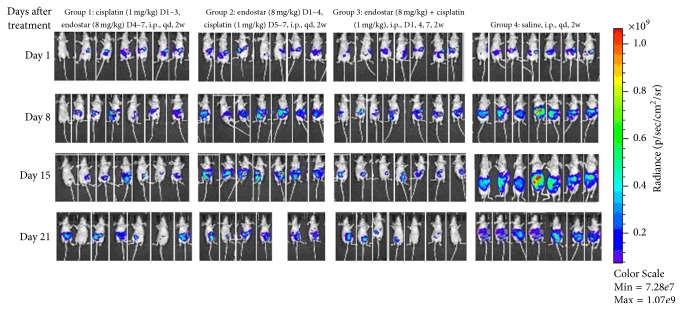
Photons analysis of the four experimental groups.

By using the in vivo imaging processing software, tumor growth curves were plotted according to the number of photons per measurement (Figure 2). On days one and eight, there were no significant differences among the four groups (*P* > 0.05).

**Figure 3:**
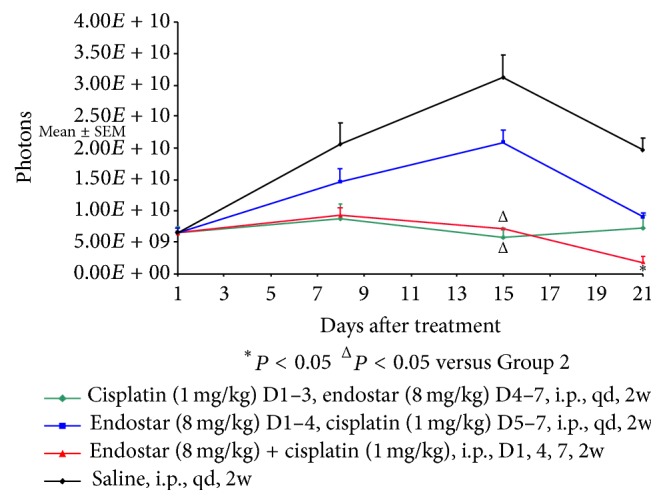
Photons analysis of the experimental groups by repeated measures process of the general linear model.

On days 15 and 21, the number of cancer cell photons in groups 1, 2, and 3 was all less than that in the control group (*P* < 0.01). On day 15 (after two weeks of treatment), the count of cancer cell photons in group 3 (the simultaneous combination of endostar with the cisplatin group) and group 1 (the sequential cisplatin-endostar group) was less than that in group 2 (the endostar sequential cisplatin group) (*P* < 0.05). On day 21, the count in group 3 continued to be less than that in group 2 (*P* < 0.05). However, there was no significant difference between the former two groups (*P* > 0.05). On day 21, the number of cancer cell photons in group 3 (simultaneous combination of endostar with the cisplatin group) continued to be less than that in group 2 (the sequential endostar-cisplatin group) (*P* < 0.05) (Figure 3).

### 3.4. MVD and LVD

**Figure 4:**
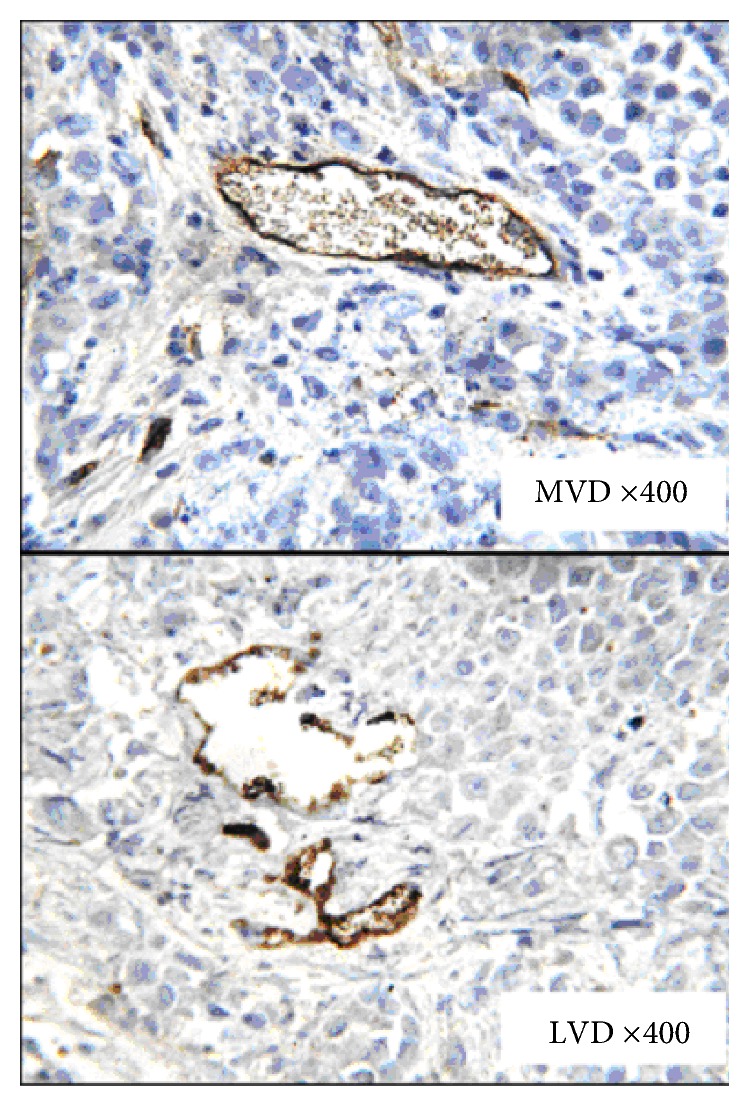
Immunohistochemical analysis of MVD and LVD in cancer cells from transplanted peritoneal tumor nodules (CD34-positive blood vessels and D2-40-positive lymphatic vessels in peritoneal nodules).

**Figure 5:**
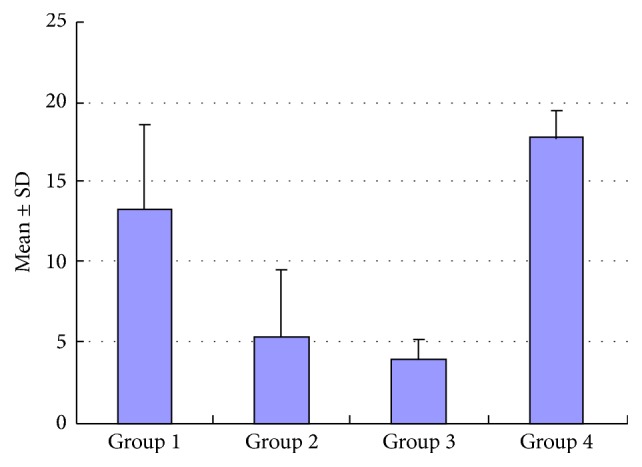
Immunohistochemical analysis of MVD in peritoneal tumor nodules (mean ± SD).

The density of blood vessels and lymphatics was measured by CD34 and D2-40 staining (Figure 4). There were differences in MVD among the four groups (*P* < 0.01). The group rank was as follows: group 4 > group 1 > group 2 (*P* < 0.01); and there was no difference between group 2 and group 3 (*P* > 0.05) (Figure 5). However, there were no statistical differences in LVD among the four groups (*P* > 0.05).

## 4. Discussion

Due to the reduction in blood supply, cells may not be conducive to chemotherapeutic drug activity. Controlling the timing of administration of the antiangiogenic agent could normalize blood vessels, and chemotherapy during the window period could make it evenly distributed, so as to achieve the maximum effect of chemotherapy. Another rationale for the administration of chemotherapy followed by antivascular treatment is that chemotherapy could minimize tumor burden and reduce VEGF prior to exposure to antiangiogenic therapy, inhibiting blood vessel and tumor growth [24]. Although chemotherapy drugs have different mechanisms of action and doses, the sequence and timing of chemotherapy and antiangiogenic agents need to be tailored to the agents that would be used. The method to determine the lowest effective dose and the window period for the normalization of tumor blood vessels for sequential chemotherapy and antiangiogenic therapy remains unclear. In animal models of solid tumors, the optimal therapeutic window of endostar combined with platinum or paclitaxel has been reported to be approximately 4–6 days by some authors [25] and 3–7 days by other authors [24]. During that time, the tumor normalization of microvascular matures and hypoxia partially improves. Hence, within the time window of antiangiogenic therapy and chemotherapy, the antitumor effect was found to be the most significant. After that time, antiangiogenic therapy becomes excessive, resulting in the gradual reduction in chemotherapy drug concentration inside the tumor tissue. Patients with solid tumors treated with endostar at 4–6 days prior to chemotherapy have been shown to have had significantly improved antitumor effects. These data provides a basis for the proposed sequential use of endostar with cisplatin.

Mouse subcutaneous tumor growth can be easily monitored by caliper measurements. However, in pleural metastasis models, it is difficult to measure tumor growth continuously and evaluate responses to treatment. In vivo optical imaging by bioluminescence or fluorescence can provide real-time observation of gene and cell marker activities in a living animal body. This method has become increasingly used in medical and biological research. Green/red fluorescent protein (GFP/RFP) [26–29] has been widely used because it is suitable for single cell in vitro and in vivo functional testing, and its use could have improved the current study in terms of cellular detail. However, luciferase (Luc) has a longer emission wavelength than GFP and is, therefore, more suitable for examination of small lesions in deep tissue tumors, distant metastases in living body, and monitoring dynamic changes. Compared with orthotopic transplantation models, NUGC-4-Luc peritoneal subcutaneous implants have been reported to better simulate the transfer process in the body and the biological behavior of peritoneal metastasis [30]. The fundamental advantage of Luc-labeled cell in vivo imaging is that the luciferase gene is integrated into the chromosome. When cells divide and differentiate, the luciferase is sustained and stably expressed. The luciferin substrate provided by intraperitoneal or intravenous injection would generate luminescence within minutes. However, this enzyme would only produce luminescence within living cells; and the emitted light intensity is linearly related to the number of labeled cells. By measuring the number of photons, the number of cancer cells can be calculated quantitatively. Currently, Luc-labeled cells have been used previously for solid tumor studies [31] and in the pleural effusion studies of Matsumoto et al. [23], but not in ascites. In the present study, we established the gastric ascites tumor model in nude mice of NUGC-4-Luc cell lines and performed in vivo bioluminescence imaging. Using in vivo imaging techniques to explore the mechanism of growth and metastasis of gastric ascites tumors and the development of anticancer drugs, we provided a stable and reliable, intuitive, convenient, and sensitive animal model. Compared with orthotopic transplantation models, NUGC-4-Luc peritoneal subcutaneous implants have been reported to better simulate the transfer process in the body and the biological behavior of peritoneal metastasis [30]. The results revealed that 15 days after treatment, cisplatin sequential endostar and combination group were superior to endostar sequential cisplatin in killing cancer cells, while the combined group was still superior to endostar sequential cisplatin group 21 days after treatment.

MVD and LVD are important indicators to evaluate tumor blood vessels and lymphatics. The relationship between MVD, LVD, and poor prognosis of cancer patients has been demonstrated in a variety of tumors. The study counted the number of vessels per unit area by CD34 and D2-40 marker of vascular density MVD and LVD, in order to further explore the relationship between different endostar modes and vessels. These results revealed that endostar sequential cisplatin and the combination group were superior to cisplatin sequential endostar in the inhibitory effect on endothelium. Compared to tumor angiogenesis, little attention has been given to the study of tumor lymphatic angiogenesis. The main reason for this is the fact that no specific markers of lymphatic vessels have been identified. Endostar can reduce vascular permeability, suppress the formation fluid, or inhibit lymph node metastasis in the treatment of serous cavity effusion. Basic research has shown that endostar can inhibit not only MVD, but also LVD [10]. However, the present study found no differences in LVD in peritoneal nodules among the four treatment groups, and there was no difference in the inhibition of lymphatic vessels between endostar with simultaneously and sequentially administered cisplatin. In the future, more studies on vascular morphology in malignant peritoneal effusion would be needed to determine the mechanisms of the effects of endostar and cisplatin.

In conclusion, we established the peritoneal metastasis of a gastric cancer model using NUGC-4-Luc cells and demonstrated that the intrapleural administration of endostar simultaneously and sequentially with cisplatin was a safe and effective treatment for MPE. The results of the simultaneously treated group were superior to that in the sequential groups, in terms of killing cancer cells and inhibiting vascular endothelial growth. Cisplatin-endostar sequential treatment was superior to endostar-cisplatin sequential treatment in killing cancer cells, while the latter was superior to the former in inhibiting peritoneal vascular endothelial growth. It has been suggested that molecular markers can be used to forecast peritoneal vascular endothelial changes, and functional imaging can be used to monitor the number of cancer cells and guide the rational choice of drugs, as well as the timing of administration. In the future, it may be that only molecular markers combined functional imaging techniques would be required for evaluation of treatment efficacy in malignant effusions. There are some limitations to the present study. In the short term, this cell line only formed peritoneal metastasis without involvement of other areas of the body. In addition, there was a lack of controls sacrificed at same time as the test animals and a lack of data on survival. Future research in the screening of different cell lines, peritoneal blood vessels, lymphatic vessels, and the administration of various drug concentrations and survival would be necessary to confirm these current observations. The value of this treatment technique requires confirmation in clinical trials. The future of individualized treatment remains a multidisciplinary, multicenter collaboration, and requires continued translation of basic research into clinical practice.
